# Intrarectal Administration of Adelmidrol plus Hyaluronic Acid Gel Ameliorates Experimental Colitis in Mice and Inhibits Pro-Inflammatory Response in Ex Vivo Cultured Biopsies Derived from Ulcerative Colitis-Affected Patients

**DOI:** 10.3390/ijms25010165

**Published:** 2023-12-21

**Authors:** Irene Palenca, Luisa Seguella, Aurora Zilli, Silvia Basili Franzin, Alessandro Del Re, Federico Pepi, Anna Troiani, Marcella Pesce, Sara Rurgo, Fatima Domenica Elisa De Palma, Gaetano Luglio, Francesca Paola Tropeano, Giovanni Sarnelli, Giuseppe Esposito

**Affiliations:** 1 Department of Physiology and Pharmacology “V. Erspamer”, Sapienza University of Rome, Piazzale Aldo Moro 5, 00185 Rome, Italy; irene.palenca@uniroma1.it (I.P.); aurora.zilli@uniroma1.it (A.Z.); silvia.basilifranzin@uniroma1.it (S.B.F.); alessandro.delre@uniroma1.it (A.D.R.); giuseppe.esposito@uniroma1.it (G.E.); 2 Department of Chemistry and Drug Technologies, Sapienza University of Rome, P.le Aldo Moro 5, 00185 Rome, Italy; federico.pepi@uniroma1.it (F.P.); anna.troiani@uniroma1.it (A.T.); 3 Department of Clinical Medicine and Surgery, University of Naples “Federico II”, 80131 Naples, Italy; mapesc@hotmail.com (M.P.); sararurgo91@gmail.com (S.R.); sarnelli@unina.it (G.S.); 4 Department of Molecular Medicine and Medical Biotechnologies, Centro Ingegneria Genetica-Biotecnologie Avanzate s.c.a rl, 80131 Naples, Italy; fatimadomenicaelisa.depalma@unina.it; 5 Endoscopic Surgery Unit, Department of Medical and Surgical Gastrointestinal Disease, Federico II University of Naples, 80131 Naples, Italy; gaetano.luglio@unina.it (G.L.); fpt.tropeano@gmail.com (F.P.T.)

**Keywords:** experimental mouse colitis, DNBS, intrarectal adelmidrol/hyaluronic acid gel administration, medical device, human biopsies

## Abstract

Improving clinical outcomes and delaying disease recrudescence in Ulcerative Colitis (UC) patients is crucial for clinicians. In addition to traditional and new pharmacological therapies that utilize biological drugs, the development of medical devices that can ameliorate UC and facilitate the remission phase should not be overlooked. Drug-based therapy requires time to be personalized and to evaluate the benefit/risk ratio. However, the increasing number of diagnosed UC cases worldwide necessitates the exploration of new strategies to enhance clinical outcomes. By incorporating medical devices alongside pharmacological treatments, clinicians can provide additional support to UC patients, potentially improving their condition and slowing down the recurrence of symptoms. Chemically identified as an azelaic acid derivative and palmitoylethanolamide (PEA) analog, adelmidrol is a potent anti-inflammatory and antioxidant compound. In this study, we aimed to evaluate the effect of an intrarectal administration of 2% adelmidrol (Ade) and 0.1% hyaluronic acid (HA) gel formulation in both the acute and resolution phase of a mouse model of colitis induced via DNBS enema. We also investigated its activity in cultured human colon biopsies isolated from UC patients in the remission phase at follow-up when exposed in vitro to a cytomix challenge. Simultaneously, with its capacity to effectively alleviate chronic painful inflammatory cystitis when administered intravesically to urological patients such as Vessilen, the intrarectal administration of Ade/HA gel has shown remarkable potential in improving the course of colitis. This treatment approach has demonstrated a reduction in the histological damage score and an increase in the expression of ZO-1 and occludin tight junctions in both in vivo studies and human specimens. By acting independently on endogenous PEA levels and without any noticeable systemic absorption, the effectiveness of Ade/HA gel is reliant on a local antioxidant mechanism that functions as a “barrier effect” in the inflamed gut. Building on the findings of this preliminary study, we are confident that the Ade/HA gel medical device holds promise as a valuable adjunct in supporting traditional anti-UC therapies.

## 1. Introduction

Inflammatory bowel diseases (IBDs) are chronic and multifactorial illnesses characterized by remission periods and recurrent flares in which diarrhea, visceral pain, rectal bleeding/bloody stools, and weight loss are the main clinical symptoms [1]. Ulcerative Colitis (UC) and Chron’s disease (CD) are the most common forms of IBDs, and despite their uncertain etiology [2,3], according to Global Disease Burden (GBD) IBD Collaborators, UC affects millions of people worldwide (2020), and current treatments have varying levels of efficacy and adverse reactions with a refined evaluation by the clinician and patients about the best combination of the risk/benefit ratio [4]. Despite being commonly prescribed for a UC treatment, traditional long-term therapies like corticosteroids, immunosuppressants, and biologic agents can cause substantial and unpredictable side effects, negatively impacting the stability of patients [5]. It is, thus, pivotal to identify and pharmacologically characterize new drugs and/or medical devices to amplify the current tools against UC. Adelmidrol (N,N′-bis(2-hydroxyethyl-nonanediamide) is a synthetic compound with anti-inflammatory, analgesic, and antioxidant properties deriving from azelaic acid and plays interesting roles in modulating the immune response and reducing oxidative stress [6]. Oral adelmidrol administration in mice revealed powerful anti-inflammatory effects, and it has been demonstrated to reduce oxidative stress, increasing the integrity of the intestinal mucosal barrier during experimental colitis damage [7]. Many of the beneficial effects displayed by adelmidrol are linked to its intrinsic capability to increase the level of the endogenous palmitoylethanolamide (PEA) via the so-called “entourage effect” [8]. Furthermore, we recently showed that adelmidrol can effectively enhance the therapeutic approach based on PEA in various intestinal disorders. It achieves this by increasing the production and availability of PEA through its selective targeting of peroxisome proliferator-activated receptors-γ (PPAR-γ) [9]. To maximize adelmidrol’s beneficial effects, adelmidrol has been co-administered with hyaluronic acid (HA), a glycosaminoglycan of the extracellular matrix [10], and such an association has demonstrated promising benefits in controlling the inflammatory processes characteristic of pathological conditions, such as osteoarthritis [11] and spinal cord injury [12]. However, there is currently a lack of evidence regarding the potential therapeutic effect of adelmidrol/HA association in intrarectal medical device systems during colitis. Nevertheless, pharmacologically associated adelmidrol (Ade)/HA restored the urothelium tissue’s integrity in interstitial cystitis/painful bladder syndrome (IC/PBS) via topic intravesical administration. In this way, such a sterile medical device can topically reduce inflammation in the urothelium, displaying a synergistic anti-inflammatory effect in the bladder [13]. Based on this background, the aims of the present study were to (i) investigate the mechanical effect of the association of Ade/HA administered via an intrarectal route in a mouse model of DNBS-induced colitis, testing the effect of HA 0.1% and adelmidrol 2% gel formulation on the amelioration of colon inflammation during both the acute (3rd day) and late (7th day) resolution of colitis. (ii) In this context, we evaluated the inhibitory effect of HA on the absorption of adelmidrol, addressing its function through a mechanical action in situ that promotes intestinal homeostasis and exhibits inhibitory effects against the degradation of HA by hyaluronidase-1 (HYAL-1). (iii) From a translational perspective, to confirm the protective effect of Ade/HA gel, we also assessed its protective effect in human biopsies isolated from patients diagnosed with UC in the remission phase. The biopsies were treated with cytomix (lipo-polisaccharide (LPS), interferon-γ (IFN-γ), and tumor necrosis factor-α (TNF-α)) in vitro to re-activate the inflammatory process. The results of the study demonstrated the protective action of the Ade/HA formulation, leading to a reduction in colon inflammation and a potential decrease in mucosal damage and permeability. Overall, this study suggests that the mechanical action of the Ade/HA gel formulation makes it a promising medical device for the prevention of mucosal damage and related inflammatory complications for the treatment of colitis.

## 2. Results

### 2.1. The Endoscopic Evaluation, Colon Length Measurement, Spleen Weight and DAI Score Showed That Ade/HA Intrarectal Administration Ameliorated Both the Acute and Post-Remission Phase of DNBS-Induced Colitis

**Figure 1:**
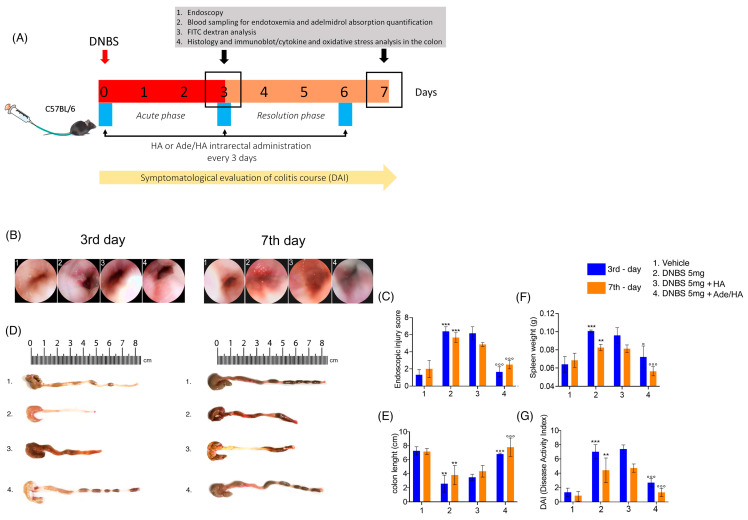
Experimental design and the ameliorative effect of intrarectal Ade/HA gel administration on DNBS-induced colitis in mice. Synoptic frame showing (**A**) the experimental plan of colitis induction and relative treatments in mice and relative time points for the DAI course, treatments, blood collection, and endoscopic procedures, and the post-mortem processing of the samples. The figure shows the endoscopic (**B**) images of the mice colon on the 3rd and 7th days after DNBS enema and (**C**) the respective endoscopic injury score evaluation; (**D**,**E**) representative post-mortem colon length examination and quantification. (**F**) Spleen weight quantification in mice on the 3rd and 7th day after DNBS enema and (**G**) DAI course in the experimental intervals. Results are expressed as the mean ± SD of *n* = 5 experiments *** *p* < 0.001 vs. *vehicle*; ** *p* < 0.01 vs. *vehicle*; °°° *p* < 0.001 vs. *DNBS*; ° *p* < 0.001 vs. *DNBS*.

The endoscopic evaluation of mouse rectal mucosa (rectoscopy) revealed that the administration of DNBS caused a significant increase in colitis features measured as the injury score on the third day after DNBS stimulus (+392% *** p < 0.001 vs. *vehicle*) [Figure 1]. Such increased damage was slightly reduced in the resolving phase of DNBS-induced colitis on the seventh day, but it remained significantly higher in comparison to the corresponding vehicle (+185% *** p < 0.001 vs. *vehicle*) [Figure 1]. Every 72 h, the intrarectal administration of Ade/Ha gel markedly impacted the colitis’ course since it resulted in a significant reduction in injury score values both on the 3rd day (−75% *°°° p* < 0.001 vs. *DNBS*) and on the 7th day (−55% *°°° p* < 0.001 vs. *DNBS*) [Figure 1]. Conversely, no relevant impact on mouse mucosa protection was observed following HA gel treatment alone. In fact, in the same experimental conditions, histological damage scores were not affected significantly on the 3rd (−4.6% vs. *DNBS*) or 7th day (−10% vs. *DNBS*) [Figure 1]. Our data show that DNBS treatment caused a marked and significant decrease in the colon length with respect to the vehicle group, and colon shortening was particularly evident on the 3rd day (−70% ** p< 0.01 vs. *vehicle*) and was still observable on the 7th day (−50% ** p < 0.01 vs. *vehicle*) [Figure 1]. In parallel to the endoscopic evaluation, Ade/HA resulted in a marked rescue of colon length in the two time points on the 3rd (+169% *°°° p* < 0.001 vs. *DNBS*) and 7th day (+100% *°°° p* < 0.001 vs. *DNBS*), whereas no significant effect was detected following HA treatment alone in both the acute (+9.4% vs. *DNBS*) and remission phase (+10% vs. *DNBS*) of the colitis [Figure 1]. As expected, the DNBS challenge caused significant spleen weight in both the acute (+63% *** p < 0.001 vs. *vehicle*) and remission phase (+25% ** p < 0.01 vs. *vehicle*); these data were accompanied by a marked increase in the DAI score on the 3rd day (+305% *** *p* < 0.001 vs. *vehicle*) and 7th day (+166% ** p < 0.01 vs. *vehicle*). The intrarectal administration of Ade/HA gel every 72 h resulted in a significant improvement in all the parameters. It reduced spleen weight on the 3rd day (−34% *° p* < 0.05 vs. *DNBS*) and 7th day (−42% *°°° p* < 0.001 vs. *DNBS*) and attenuated the DAI score on the 3rd (−64% *°°° p* < 0.001) and 7th days (−77% *°°° p* < 0.001) vs. *DNBS.* No significant spleen weight reduction was observed following the HA treatment on the 3rd (−10% vs. *DNBS*) and 7th days (−3% vs. *DNBS*); the DAI score for the same intervals was (+2% vs. *DNBS*) and (−2.4% vs. *DNBS)*, respectively [Figure 1].

### 2.2. Intrarectal Ade/HA Administration Reduced ZO-1 and Occludin Loss Induced by DNBS in Both Acute and Remission Phase of Colitis

**Figure 2:**
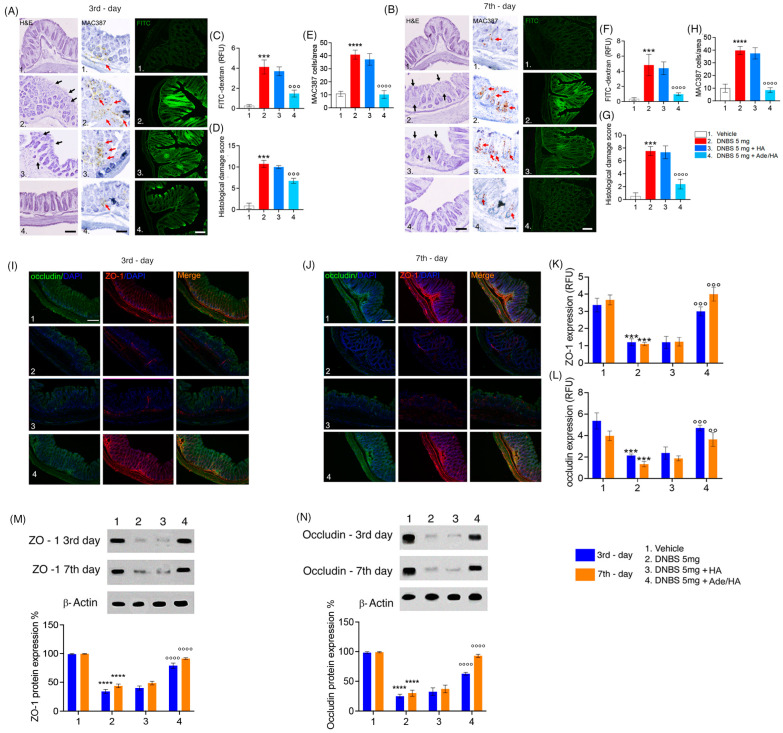
Intrarectal Ade/Ha gel administration ameliorates DNBS-induced histological damage and improves mucosal integrity by rescuing ZO-1 and occludin expression in mice. Synoptic frame showing the protective effect of Ade/Ha on mice mucosa in the acute and resolution phase of colitis induced by DNBS enema. The figure at the top shows the histological evaluation using hematoxylin–eosin staining and colon permeability via FITC-dextran immunofluorescence analysis carried out on the mice colon on the 3rd (**A**) and 7th day (**B**), respectively, after DNBS enema in the presence of different treatments. Respective quantification of both FITC-dextran relative fluorescence units (RFU), the histological damage score, and immunohistochemical expression of MAC387-positive cell on the 3rd (**C**–**E**) and 7th day (**F**–**H**). Magnification 20×; Scale bar 100 μm. The middle panel shows the immunofluorescence analysis of occludin and ZO-1 and their merged expression, respectively, on the 3rd and 7th day after DNBS enema (**I**,**J**) and respective quantification by RFU (**K**,**L**)—Magnification 20×; Scale bar 100 μm. In the lower panel, the figure shows the immunoblot expression and respective quantifications of ZO-1 (**M**) and Occludin (**N**), respectively, on the 3rd and 7th day after the DNBS enema. Results are expressed as the mean ± SD of *n* = 5 experiments **** *p <* 0.0001 vs. vehicle; *** *p* < 0.001 vs. vehicle; °°°° *p <* 0.0001 vs. DNBS; °°° *p* < 0.001 vs. DNBS; °° *p* < 0.01 vs. DNBS. Black arrows indicate areas of crypt loss and inflammation. Red arrows indicate a positive cluster of resident macrophages expressing the MAC387 protein.

After the DNBS challenge, significant mucosal damage was observed at both time points according to the macroscopic evaluation carried out before. The histological damage score was significantly increased in the DNBS vs. the vehicle group on the 3rd day (+800% *** p < 0.001 vs. *vehicle*) and was still evident on the 7th day (+567% *** p < 0.001 vs. *vehicle*). A significant increase in the infiltration of macrophages labeled with MAC387 was evident in the DNBS vs. vehicle group on the 3rd (+378% **** p < 0.0001 vs. *vehicle*) and 7th days (+398% **** p < 0.0001 vs. *vehicle*). In parallel to this, the absorption of FITC dextran dye was markedly increased across the colitis mucosa on the 3rd (+700% *** p < 0.001 vs. *vehicle*) and 7th days (+760% *** p < 0.001 vs. *vehicle*) following the DNBS challenge, confirming the overall damage of the mouse mucosa revealed by histological analysis [Figure 2]. No significant protective effect was observed by HA administration in the same experimental conditions. Conversely, there was a significant and consistent reduction in both mucosal damage and FITC-dextran permeability following the intrarectal administration of Ade/HA. This effect was evident for a significant reduction in the histological damage score of DNBS-induced tissue injury on the 3rd day (compared to *DNBS*, −53% °°° *p <* 0.001) and 7th day (compared to *DNBS*, −80% °°° *p <* 0.001). Furthermore, a significant reduction in MAC387-positive cell counts in the mucosa on the 3rd day (−75% °°°° *p <* 0.0001 vs. *DNBS*) and 7th day (−79% °°°° *p <* 0.0001 vs. *DNBS*) was detected. Moreover, Ade/HA treatment led to a significant decrease in the transmucosal passage of FITC dye on the 3rd day (compared to DNBS, −63% °°° *p <* 0.001) and 7th day (compared to DNBS, −78% °°° *p <* 0.001) [Figure 2]. Immunofluorescence and immunoblot analysis confirmed that the DNBS challenge resulted in a significant reduction in ZO-1 and occludin expression, indicating a severe disruption of the gut barrier integrity in colitis. Specifically, on day 3 post-DNBS challenge, we identified a significant decrease in ZO-1 (−69% *** p < 0.001 vs. *vehicle*) and occludin (65% *** p < 0.001 vs. *vehicle*). This effect persisted to a lesser but still significant extent during the resolution phase of colitis on day 7 post-DNBS challenge, with reductions in ZO-1 (77% *** p < 0.001 vs. *vehicle*) and occludin (75% *** p < 0.001 vs. *vehicle*). Immunoblot analysis confirmed these findings, showing significant reductions in ZO-1 and occludin expression compared to the DNBS challenge, both in the acute phase (−70% **** p < 0.0001 and −80% **** p < 0.0001 vs. *respective vehicle groups* for ZO-1) and resolution phase of colitis (−75% **** p < 0.0001 and −70% **** p < 0.0001 vs. *respective vehicle groups* for occludin). HA alone did not restore the expression of tight junctions in time point intervals via the immunofluorescence of ZO-1 on the 3rd or 7th day after the DNBS challenge (+0.5% vs. *DNBS* and +1% vs. *DNBS*, respectively). In parallel to this, no HA effect was detected for occludin both on the 3rd and 7th day after the DNBS challenge (+1% and +2% vs. *DNBS*, respectively). Such data matched with immunoblot analysis showing that HA alone did not impact both ZO-1 expression on the 3rd (+2% vs. *DNBS*) and 7th days after the DNBS challenge (+2.2% vs. *DNBS*), and nor did it result in the ability to modify occludin expression in the same interval range (+1% vs. *DNBS* at 3rd day and +2% vs. *DNBS* at 7th day after DNBS challenge, respectively). Immunofluorescence analysis demonstrated that Ade/HA gel intrarectal administration significantly preserved the mucosal barrier by improving both ZO-1 and occludin expression on the 3rd (+190% *°°° p* < 0.001 for ZO-1 and +115% *°°° p* < 0.001 for occludin vs. *respective DNBS groups*) and 7th day (+290% *°°° p* < 0.001 for ZO-1 °°° *p <* 0.001 and +105% °° *p* < 0.01 for occludin vs. *respective DNBS groups*). According to these data, immunoblot analysis confirmed that Ade/HA gel caused the significant restoration of both ZO-1 and occludin expression on the 3rd (+180% *°°°° p* < 0.0001 for ZO-1 and +135% *°°°° p* < 0.0001 for occludin vs. *respective DNBS groups*) and 7th day (+100% for ZO-1 *°°°° p* < 0.0001 and +105% *°°°° p* < 0.0001 for occludin vs. *respective DNBS groups*) [Figure 2].

### 2.3. Intrarectal Ade/HA Administration Reduced DNBS-Induced Proinflammatory Response, Oxidative Stress and Endotoxemia without Affecting Endogenous PEA Level and in Absence of Systemic Adelmidrol Absorption

**Figure 3:**
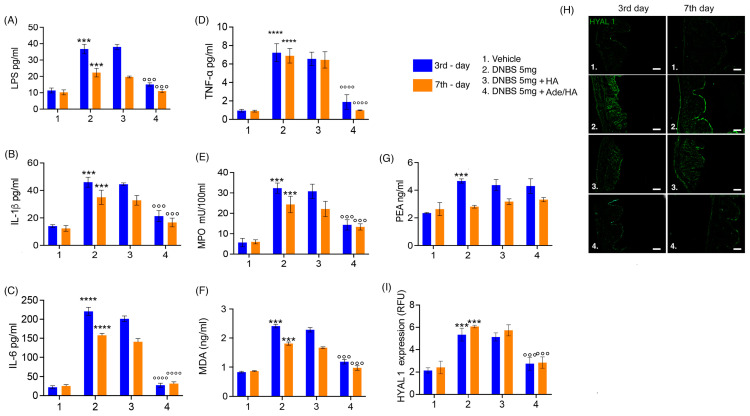
Ade/Ha intrarectal administration reduces endotoxemia and oxidative stress without interfering with the PEA level and inhibiting HYAL-1 expression in the mice colon during DNBS-induced colitis. The figure shows the effect of the intrarectal administration of Ade/HA on endotoxemia (**A**) and IL-1*β* release (**B**) in the plasma affected by colitis on the 3rd and 7th day after DNBS challenge; the Figure also shows (**C**) IL-6 release, (**D**) TNF-α release, (**E**) MPO, (**F**) MDA, (**G**) and the quantification of PEA. On the right panel, (**H**) the immunofluorescence analysis of HYAL1 expression on the 3rd and 7th day after the DNBS challenge in the presence of different treatments and (**I**) the relative quantification expressed as RFU are presented. Magnification: 10×; scale bar: Results are expressed as the mean ± SD of *n* = 5 experiments **** *p* < 0.0001 vs. vehicle; *** *p* < 0.001 vs. vehicle °°°° *p* < 0.0001 vs. DNBS; °°° *p* < 0.001 vs. DNBS.

Consistent with the expectations, the DNBS challenge elicited a pronounced pro-inflammatory response and oxidative stress in the mouse colon, as evidenced by significant increases in IL-1β (+138% *** p < 0.001 vs. *vehicle*), IL-6 (+1005% **** p < 0.0001 vs. *vehicle*) and TNF-α (+766% **** p < 0.0001 vs. *vehicle*), neutrophil infiltration indicated by up-regulation of MPO (+540% *** p < 0.001 vs. *vehicle*) and the oxidative stress induction manifested by a substantial rise in the lipid peroxidation marker malondyaldheide (MDA) (+158% *** p < 0.001 vs. *vehicle*) during the acute phase of colitis [Figure 3]. Moreover, the previously mentioned disruption of gut mucosal integrity was accompanied by a significant increase in endotoxemia during the acute phase of colitis (+218% *** p < 0.001 vs. *vehicle*) [Figure 3]. Even during the resolution phase of colitis, although to a slightly lesser extent, all the pro-inflammatory parameters remained markedly increased compared to the respective vehicle group. Notably, there was a significant up-regulation of IL-1β (+131% *** p < 0.001 vs. *vehicle*), IL-6 (+632% **** p < 0.0001 vs. *vehicle*) and TNF-α (+861% **** p < 0.0001 vs. *vehicle*), MPO (+177% *** p < 0.001 vs. *vehicle*), MDA accumulation (+90% *** p < 0.001 vs. *vehicle*) and LPS detection (+110% *** p < 0.001 vs. *vehicle*) in the serum [Figure 3]. According to the previously obtained data, no significant effect of HA alone was detected at both time points, clearly showing no significant improvement in all pro-inflammatory, oxidative, and endotoxemia parameters in comparison to the DNBS groups. Conversely, the intrarectal administration of Ade/HA was able to produce a significant reduction in IL-1β (−48% *°°° p* < 0.001 vs. *DNBS*), IL-6 (−87% *°°°° p* < 0.0001 vs. *DNBS*), TNF-α (−74% *°°°° p* < 0.0001 vs. *DNBS*), MPO (−58% *°°° p* < 0.001 vs. *DNBS*), MDA production (−55% *°°° p* < 0.001 vs. DNBS) and endotoxemia (−66% *°°° p* < 0.001 vs. *DNBS*) in the acute phase of colitis, and such an effect was still preserved in the resolution phase of the experimental colitis, whereas a marked inhibition of IL-1β (−51% *°°° p* < 0.001 vs. *DNBS*), IL-6 (−80% *°°°° p* < 0.0001 vs. *DNBS*), TNF-α (−86% *°°°° p* < 0.0001 vs. *DNBS*), MPO (−52% *°°° p* < 0.001 vs. *DNBS*), MDA production (−44% *°°° p* < 0.001 vs. *DNBS*) and endotoxemia (−45% *°°° p* < 0.001 vs. *DNBS*) was reported [Figure 3]. Moreover, the DNBS challenge caused a massive increase in HYAL-1 expression on the 3rd day (152% *** *p <* 0.001 vs. DNBS group), and such an effect was still preserved on the 7th day after colitis induction (+172% *** p < 0.001 vs. *DNBS group*). In full agreement with the previous results, HA alone did not show any significant effect on HYAL-1 expression in the tissue both in the acute (−5% vs. *DNBS group*) and resolution phase (−4% vs. *DNBS group*) of colitis. On the contrary, Ade/HA administration resulted in a significant HYAL-1 reduction on the 3rd (−58% *°°° p* < 0.001 vs. *DNBS group*) and 7th (−60% *°°° p* < 0.001 vs. *DNBS group*) day after the DNBS challenge. Our data revealed that after the DNBS challenge, endogenous PEA levels were elevated on the 3rd day following colitis induction (+91% *** p < 0.001 vs. *vehicle*), but neither HA administration (+9% vs. *DNBS*) nor, more interestingly, Ade/HA administration (+8% vs. *DNBS*) significantly altered PEA accumulation during the acute phase of colitis. Interestingly, the DNBS-induced increase in PEA levels was no longer present on the 7th day of colitis (+2% vs. *vehicle*), and, once again, neither HA (−0.6% vs. *DNBS*) nor Ade/HA (−1% vs. *DNBS*) administration had a significant effect on PEA levels. This suggests that the protective effects of the intrarectal administration of the Ade/HA gel were independent of any potentiation of endogenous PEA effects [Figure 3]. Furthermore, our results demonstrate that the intracolonic administration of adelmidrol plus HA gel did not result in the detectable systemic absorption of this molecule (Table 1).

### 2.4. Ade/HA Inhibited Cytomix-Induced Pro-Inflammatory Response in Cultured Human UC-Deriving Biopsies without Interfering with in Ex-Vivo PEA Production

**Figure 4:**
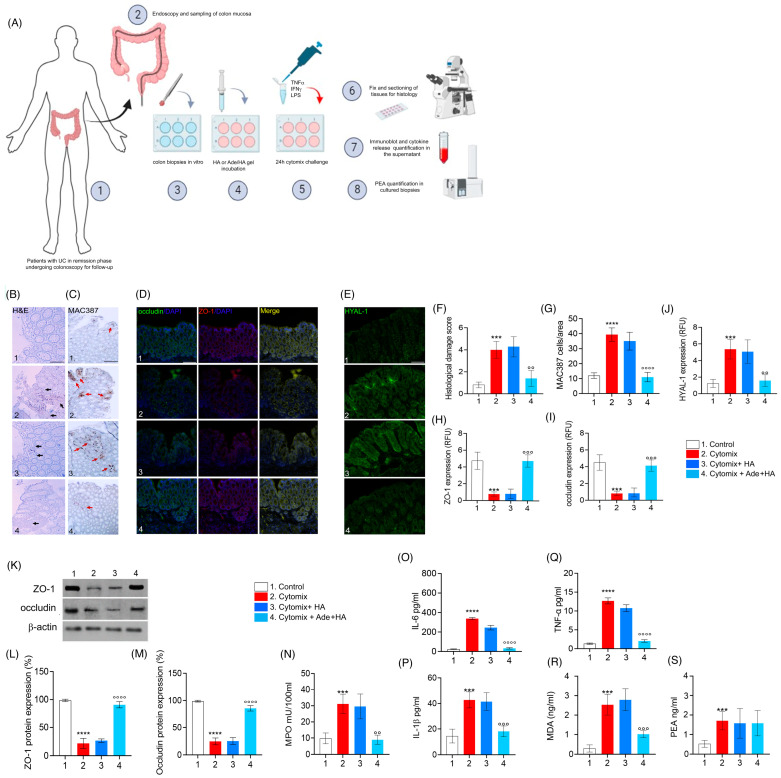
The in vitro incubation of Ade/HA gel ameliorates the pro-inflammatory status in remission phase-isolated UC biopsies stimulated with cytomix without affecting PEA release. The synoptic frame is shown at the top (**A**) with the experimental plan and procedures for ex vivo experiments in human biopsies isolated by UC patients in the remission phase having undergone follow-up colonoscopy in vitro-challenged with cytomix. In the middle part of the figure panel, we show, from left to the right, the protective effect of Ade/HA on histological damage via hematoxylin/eosin analysis (**B**), the immunohistochemical expression of MAC387 positive cells (**C**), the rescue of occludin and ZO-1, (**D**) the inhibition of HYAL-1 immunofluorescence analysis (**E**) and the respective quantification of the histological damage score, cell count and RFU (**F**–**J**) in human colon biopsies exposed to the cytomix challenge for 24 in vitro after their isolation. Magnification 20×; Scale bar: 100 μm. In the lower panel, the figure shows the effect of Ade/Ha on ZO-1 and occludin expression via immunoblot analysis (**K**) and its relative quantification (**L**,**M**); in the same experimental conditions, the effect of Ade/Ha and other treatments were evaluated on (**N**) MPO, (**O**) IL-6, (**P**) IL-1*β*, (**Q**) TNF-α, (**R**) MDA and (**S**) PEA quantification in cultured biopsies. Results are expressed as the mean ± SD of *n* = 5 experiments *** *p* < 0.001 vs. *Control*; **** *p* < 0.0001 vs. *Control*; °° *p* < 0.01 vs. *Cytomix* °°° *p* < 0.001 vs. *Cytomix*. °°°° *p* < 0.0001 vs. *Cytomix*. Black arrows indicate areas of crypt loss and inflammation. Red arrows indicate a positive cluster of resident macrophages expressing the MAC387 protein.

When biopsies deriving from patients with UC in the remission phase underwent a challenge with cytomix (TNF-α, INF-γ, LPS) for 24 h, a consistent pro-inflammatory response was stimulated in vitro and a significant increase in the histological damage score was observed ex vivo (+471% *** p < 0.001 vs. *control*), including a significant increase in MAC387-positive cell infiltration (+328% **** p < 0.0001 vs. *control*) as well as a marked decrease in both ZO-1 (−87% *** p < 0.001 vs. *control*) and occludin (−77% *** p < 0.001 vs. *control*) expression observed via immunofluorescence [Figure 4]. Such data were confirmed using immunoblot analysis (−85% **** p < 0.0001 vs. *control* for ZO-1 and −88% **** p < 0.0001 vs. *control* for occludin, respectively) [Figure 4]. In the same experimental conditions, the cytomix stimulation caused a consistent increase in HYAL-1 expression in cultured biopsies (+266% **** p < 0.001 vs. *control*). Accordingly, to in vivo-obtained data, the cytomix challenge also resulted in a parallel increase in tissue IL-1β (+116% *** p < 0.001 vs. *control*), IL-6 (+1425%**** p < 0.0001 vs. *control*), TNF-α (+951% **** p < 0.0001 vs. *control*), MPO (+200% **** p < 0.0001 vs. *control*), and oxidative stress via MDA accumulation (+400% *** p < 0.001 vs. *control*) in cultured bioptic samples [Figure 4]. Treatment with HA alone did not lead to a detectable reduction in any of the observed parameters. This was evident from the lack of significant effects on histological damage, occluding, ZO-1, and HYAL-1 expression , as well as the absence of a reduction in IL-1β, MPO and MDA accumulation in the biopsy’s milieu [Figure 4].

As expected, Ade/HA gel markedly reduced the pro-inflammatory response evoked by cytomix incubation ex vivo, and a significant reduction in the histological damage score (−60% *°° p* < 0.01 vs. *cytomix group*), MAC387-positive cell count (−72% *°°°° p* < 0.0001 vs. *cytomix group*), as well as a consistent rescue of both ZO-1 (+350% *°°° p* < 0.001 vs. *cytomix group*) and occludin (+300% *°°° p* < 0.001 vs. *cytomix group*) via immunofluorescence analysis, was in agreement with immunoblot analysis whereas both ZO-1 (+305% *°°°° p* < 0.0001 vs. *cytomix group*) and occludin (+290% *°°°° p* < 0.0001 vs. *cytomix group*) were markedly rescued by Ade/HA treatment [Figure 4]. Ade/HA incubation caused a marked decrease in cytomix-induced HYAL-1 up-regulation (−70% *°° p* < 0.01 vs. *cytomix group*), and, at the same time it accounted for an IL-1β (−55% *°°° p* < 0.001 vs. *cytomix group*), IL-6 (−90% *°°°° p* < 0.0001 vs. *cytomix group*), TNF-α (−84% *°°°° p* < 0.0001 vs. *cytomix group*), MPO (−70% *°° p* < 0.01 vs. *cytomix group*) and MDA accumulation decrease (−60% *°°° p* < 0.001 vs. *cytomix group*) in comparison with the cytomix group [Figure 4]. Consistent with our in vivo findings, the inflammatory response induced by cytomix incubation in the bioptic samples led to a significant increase in PEA release (+118% *** p < 0.001 vs. *control group*). Importantly, none of the treatments had an impact on PEA levels in the ex vivo setting. Our results clearly demonstrate that Ade/HA did not alter PEA levels ex vivo, further supporting the conclusion that PEA was not involved in mediating the effects of Ade/HA under our experimental conditions [Figure 4].

## 3. Discussion

One of the key challenges faced by gastroenterologists and pharmacologists currently is the quest for novel therapeutic approaches that can manage IBDs while ensuring optimal patient compliance and acceptability and considering the risk/benefit ratio [4]. It is, therefore, mandatory to investigate new therapeutic strategies that can complement existing therapies and enhance treatment outcomes over time [14]. In this regard, adelmidrol, as an analog of PEA, has garnered significant attention due to its anti-inflammatory and antioxidant properties [15,16] across various experimental conditions. In recent years, adelmidrol’s oral administration has demonstrated efficacy in the management of experimental colitis induced by DNBS in mice via inhibiting the translocation of Nuclear Factor kappa-B (NF-κB) and promoting a reduction in Intercellular Adhesion Molecule 1 (ICAM1) expression through its specific interaction with PPAR-γ receptors [7]. Although pre-clinical evidence for adelmidrol administration in mice has shown promising results, translating these findings into effective treatments for human IBDs poses a considerable challenge. The process of testing new anti-colitis drugs in humans is inherently complex and laborious. Nevertheless, with the rising global incidence of IBDs, there is an urgent demand for innovative therapeutic approaches [17]. In this regard, the use of a medical device incorporating adelmidrol and leveraging its protective potential during colitis could present a significant therapeutic approach for the treatment of UC in humans. By leveraging the benefits of adelmidrol in a localized and targeted manner, medical devices can provide a more focused and efficient delivery of the therapeutic compound. This potential medical device could be beneficial in combination with other pharmacological therapies used in the treatment of UC to improve therapy adherence, potentially lowering the dosage of the co-administered drug and thereby reducing potential systemic side effects. Furthermore, the well-known safety profile of adelmidrol and HA makes them suitable candidates for medical device applications. This formulation, with a non-pharmacological physical mechanism of action, combined with their favorable safety profiles, highlights the potential of these molecules as medical devices for the prevention and treatment of UC flareups.

In addition to its established oral anti-inflammatory properties, our research has demonstrated that the intrarectal application of Ade/HA gel leads to a significant reduction in symptom severity and colonic tissue damage in a mouse model of DNBS-induced colitis. This highlights the potential of Ade/HA gel as a promising therapeutic approach in the treatment of colitis. Our data suggest that the protective effect of such an intrarectal application increases over time. We observed that the treatment’s efficacy began during the acute phase of colitis induction on the 3rd day and reached its peak on the 7th day after the DNBS enema challenge. The Ade/HA association caused a significant improvement in colonic mucosal condition mouse colitis, exerting a topic “barrier effect” on the colon due to its capability to promote a marked inhibition of colitis-induced gut hyper-permeability, preventing a leaky gut syndrome. Our findings revealed that the Ade/HA intrarectal administration caused a significant reduction in the DNBS-induced leaky gut effect in mice, as evidenced by the prevention of FITC-dextran mucosal infiltration and a subsequent reduction in the endotoxemia level. Additionally, this treatment rescued the expression of important proteins involved in maintaining the intestinal barrier’s integrity, such as ZO-1 and occludin [18], which were negatively affected by the DNBS challenge. Such effects were, thus, accompanied by a significant increase in MAC387-positive cells, as well as a marked up-regulation of pro-inflammatory cytokines, such as IL-1β, IL-6, TNF-α, the lipid peroxidation product MDA, and a reduction in MPO levels in the inflamed colon, indicating the effectiveness of the topical administration of Ade/HA gel in reducing the extent of mucosal pro-inflammatory cells’ infiltration during colitis. In the same experimental conditions, Ade/HA gel promoted a significant antioxidant effect, reducing the amount of MDA generated by the DNBS challenge in the mouse mucosa, thus providing a synergistic effect on barrier integrity maintenance. Our data, in agreement with the formerly described antioxidant effects of adelmidrol in other inflammatory conditions [6], led us to identify such beneficial activity as the protective mechanism at the basis of the topical effect of Ade/HA gel in our experimental conditions. This is highlighted by the observation that no detectable systemic absorbance of Adelmidrol via the intrarectal administration of the Ade/HA formulation, and notably, no adelmidrol-mediated “entourage effect” on the endogenous PEA level’s up-regulation in the colon was detectable. Although recent studies have demonstrated the possible protective and anti-inflammatory role of HA during colitis [19], in our experimental conditions, this was not evident, and HA alone did not show any significant amelioration for the severity of colitis in vivo and in ex vivo experiments. Moreover, it has recently been demonstrated that intrarectal HA’s co-administration with Acetylsalicilic acid (ASA) is a very interesting approach to improving the course of colitis [20]. However, it is important to note that the studies mentioned have employed significantly higher dosages and more frequent administration compared to our experimental plan. Additionally, it is crucial to acknowledge that other studies have demonstrated the anti-IBD effects of chemically modified forms of HA, which differ from the naïve HA used in our study [21]. Furthermore, our findings revealed that that intrarectal administration of Ade/HA gel effectively reduces the expression of HYAL-1, an enzyme responsible for HA degradation, especially in the colon [22]. These results suggest the potentially synergistic effect of adelmidrol, functioning as an antioxidant agent while simultaneously inhibiting the degradation of HA in situ. By slowing down the degradation of HA, adelmidrol allows for the increased duration of the protective physical barrier that is achieved with this formulation. Mucosal biopsies deriving from patients affected by UC and challenged with cytomix were used to test Ade/HA efficacy, resulting in significant inhibition of mucosal inflammatory infiltration, with the consequent increase in the ex vivo expression of occludin and ZO-1. Consistently with the data obtained in murine colitis, Ade/HA determined a significant decrease in the release of IL-1β, TNF-α, IL-6, MPO, and MDA accumulation in cytomix-stimulated tissues, and these effects were accompanied by a significant reduction in the expression of HYAL-1. Also, in this case, no significant variation in terms of PEA release by Ade/HA gel treatment was observed in vitro; this confirmed the “virtually” independent PEA release via Ade/HA as having an “entourage” effect capable of the observed anti-inflammatory activity in human biopsies.

Although the topical application of 5-ASA is commonly used for treating distal colitis, several studies have demonstrated that the effectiveness of in vitro 5-ASA is directly linked to its dose and tissue concentration, which, in turn, drive clinical response [23]. In this context, our data highlight, for the first time, that the intrarectal instillation of Ade/HA gel has the potential to be a new therapeutic tool against experimental colitis. This is attributed to the “mechanic” and antioxidant protective barrier effect of Ade/HA gel on the gut mucosa, introducing such a formulation as an innovative approach helpful for its clinical and fast translation. Given, in fact, the observed lack of absorption of adelmidrol in our study due to its topical effect, we are led to consider Ade/HA gel a medical device rather than a traditional drug. This classification is like its application in controlling bladder inflammation as Vessilen^®^ [13]. In conclusion, despite the undeniable successes of biological drugs in the treatment of IBDs that are increasingly performing, the potential utilization of medical devices that provide a local supportive effect to conventional UC therapies should be taken into account in optimizing therapeutic treatments. Additional clinical studies are necessary to evaluate the true efficacy of Ade/HA gel as a medical device, exploring the mechanisms underlying the here-observed “barrier” effect, and determine the optimal dosage and formulation for humans in the consideration of leaky gut as a pivotal factor in UC, the implementation of this formulation alongside traditional pharmacological therapies presents a promising avenue for improved patient care and the management of this disease.

## 4. Materials and Methods

### 4.1. Animals and Experimental Design

Eight-week-old female C57BL/8J mice (Charles River, Lecco, Italy) (*n* = 64) were used for all the experiments. The procedures included in the experimental plan were approved by Sapienza University’s Ethics Committee. Animal care followed the (International Association for the Study of Pain) IASP and European Community (EC L358/1 18/12/86) guidelines on the use and protection of animals in experimental research. Colitis was induced via a single intracolonic administration of 5 mg of DNBS (Sigma Aldrich, St. Louis, MO, USA) dissolved in 100 μL of 50% ethanol (EtOH) (Sigma Aldrich, St. Louis, MO, USA) and saline (Thermo Fisher Scientific, Waltham, MA, USA) [24], whereas the vehicle group received a single intracolonic administration of saline and EtOH at 100 μL. EtOH was used as a means to effectively break the intestinal barrier and enable the interaction of DNBS with colon tissue proteins, thereby triggering the host’s innate and adaptive immune responses. Overnight-fasted mice were treated with DNBS on day 0 through a flexible catheter (Hugo-Sachs Elektronik, March, Germany) rapidly inserted approximately 3 cm from the anus without anesthesia. A total of 100 μL of the DNBS solution was introduced slowly into the colon–rectal tract and the animals were kept slightly reclined throughout the procedure. Therefore, the mice were returned to their cages and placed overnight on a heating pad to facilitate recovery. The progression of experimental colitis, and the efficacy of treatments was assessed both on the acute (3rd) day and in the remission phase (7th day) post DNBS enema. Mice were randomly divided into the following groups (*n* = 16 each): (i) vehicle (receiving 100 μL sterile saline); (ii) DNBS (colitis group, receiving 100 μL of DNBS at 5 mg in EtOH 50% sterile saline); (iii) HA (100 μL of 0.1% sodium hyaluronate saline every 72 h starting on day 0 until day 7; (iv) Adelmidrol/HA gel (100 μL HA 0.1% plus 2% adelmidrol) every 72 h starting on day 0 until day 7.

### 4.2. Patients and Tissue Culture Treatments

Recto-sigmoid mucosal biopsies were obtained from patients with a diagnosis of distal UC. Patients were considered in clinical and biochemical remission and underwent colonoscopy for screening (*n* = 8, median age: 32 years range 25–40; 4 female). Patients were asked to stop any pharmacological treatment during the 2 weeks preceding the endoscopy; exclusion criteria were (i) a history of cancer; (ii) the use of an immunosuppressant, anti-platelet, or anti-coagulant drugs; (iii) significant uncontrolled comorbidity. Patients gave their written informed consent, and the protocol was approved by the Ethics Committee of the Federico II University of Naples.

Four mucosal biopsies obtained from the recto-sigmoid region were used. In brief, specimens were immediately placed in 24-well plates and cultured in Dulbecco-Modified Eagle’s Medium (DMEM) (Sigma, Milan, Italy) supplemented with 5% fetal bovine serum, 2 mmol/l of glutamine, 100 U/I of penicillin, and 100 μg/mL of streptomycin (Biowhittaker, Milan, Italy) at 37 °C in 5% CO_2_/95% air for 24 h, as previously described [25,26,27]. To mimic active inflammation, we used a protocol previously reported by our group [28], comprising the incubation of isolated biopsies in a pro-inflammatory Cytomix constituted by LPS (10 μg/mL), plus IFN-γ (300 U/mL) and TNF-α (100 U/mL) (all from Sigma, Milan, Italy), in the presence or absence of 0.1% of HA or 2% Adelmidrol plus 0.1% HA gel, (Ade/HA) (both from Epitech group, Saccolongo-Padua, Italy) for another 24 h before being addressed to a histological, immunofluorescence and immunoblot or cytokine assay as indicated in the experiment [Figure 4A] and as described subsequently.

### 4.3. Endoscopic Procedures in Mouse and Endoscopic Damage Score Evaluation

For endoscopic procedures, *n* = 4 mice from each experimental group on both the 3rd and 7th day after the DNBS stimulus underwent anesthesia via the intraperitoneal injection of ketamine at 100 mg/mL and xylazine at 20 mg/mL [29]. The experimental endoscopy of the rectum and colon was performed using a bronchoscopy adapted for small rodent use (Karl Storz, Tuttlingen, Germany). The animals were fasted 24 h before endoscopy and underwent PBS 1X enema to favor the adequate visualization of the colonic mucosa. The endoscopic frames were acquired by a color monitor and digitally recorded on a tape (CV-190 PLUS, Olympus, Segrate, Italy). The endoscopic injury score was determined according to the method described [29].

### 4.4. Colon Length, Spleen Weight and Disease Activity Index (DAI)

Depending upon the experimental design, a group of *n* = 8 mice derived from each experimental group were euthanized on days 3 and 7 after DNBS-induced colitis. Spleen weight and colon length were measured, and colonic tissues were removed to perform macroscopic, histochemical, and biochemical analyses, as described in the experimental protocol (Figure 1A). The DAI scale was used to evaluate colitis induction and progression. The DAI score was determined by two independent observers blinded to the treatments and according to the criteria proposed by Cooper et al. (1993) [30] changes in body weight (0 = none; 1 = 1 to 5%; 2 = 5 to 10%; 3 = 10 to 20%; 4 = >20%); stool consistency (0 = normal; 2 = loose; 4 = diarrhea) and rectal bleeding (0 = normal; 2 = occult bleeding; 4 = gross bleeding) were scored. The DAI score was recorded on days 3 and 7, and the results were expressed as cumulative average scores in each experimental group.

### 4.5. Histopathological Analysis of the Mouse and Human Ex Vivo Cultured Colonic Biopsies

After sacrifice, mouse distal colons were collected on the 3rd and 7th day after the DNBS challenge. Similarly, bioptic samples were cultured ex vivo and exposed to the cytomix challenge for 24 h. The samples were then fixed in 4% paraformaldehyde (PFA), sliced into 15 μm sections, and stained with hematoxylin and eosin (H&E) for a macroscopic and histopathological assessment. Histological damage was evaluated using the complex scoring system proposed by Li et al. (2017) [31] and based on the following parameters: (i) the distortion and loss of crypt architecture; (ii) the infiltration of the inflammatory; (iii) muscle thickening; (iv) goblet cell depletion; and (v) crypt absence. The slides were analyzed using a Nikon Eclipse 80i microscope (Nikon Corporation, Tokyo, Japan), and images were captured at 10× magnification using a high-resolution digital camera (Nikon Digital Sight DS-U1). The cumulative histological damage scores were expressed as average scores in each experimental group.

### 4.6. Macrophage Infiltration Immunohistochemical Assay

Tissue samples were fixed in 4% PFA, embedded in paraffin, and sectioned into 15 μm slices for subsequent immunohistochemical processing. The slices underwent heat-mediated antigen retrieval using a sodium citrate buffer, followed by incubation with anti-MAC387 (Abcam, Cambridge, UK) at room temperature (RT), as described by Thoree et al. [32], and were then subjected to detection using the horseradish peroxidase (HRP)-conjugated compact polymer system. 3,30-diaminobenzidine (DAB) was employed as chromogen. Subsequently, the slices were counterstained with hematoxylin and examined using a microscope (Optika XDS-3L4 Ponteranica, Bergamo, Italy). Images were captured at 20× magnification using a high-resolution digital camera. The quantitative analysis was performed by blinded assessors, with the results expressed as the median number of MAC387-positive cells per unit area and determined using ImageJ 1.53 software (National Institutes of Health).

### 4.7. In Vivo Assay of the Intestinal Permeability

Before being euthanized, a group of *n* = 3 mice for each treatment on the 3rd and 7th day following DNBS enema were weighed and gavaged with 1% body weight (volume to mass) of fluorescein isothiocyanate-dextran (FITC-Dextran, 4 kDa, Sigma) and were euthanized 5 h after administration. Colon samples were fixed in PFA 4% (Thermo Fisher Scientific, Waltham, MA, USA) and cryo-sectioned in 15 μm slices. These sections were analyzed with a microscope Nikon Eclipse 80i, and images were captured at 10× magnification by a high-resolution digital camera (Nikon Digital Sight DS-U1). The results were expressed as relative fluorescence units (RFU) and as arbitrary units.

### 4.8. Zonula Occludens (ZO-1) and Occludin Immunofluorescence

On day 3rd and 7th day after DNBS, enema mice were sacrificed, and the distal colon was isolated, fixed, and sectioned into 25 μm slices. In a similar way, ex vivo cultured human biopsies were sectioned in 15 μm slices. Both mouse and human sections were blocked with bovine serum albumin and subsequently stained with the rabbit anti-ZO-1 antibody (1:100 dilution *v*/*v*; Proteintech, Manchester, UK), rabbit anti-occludin antibody (1:100 dilution *v*/*v*; Novus Biologicals, Abingdon, UK) or mouse anti-HYAL1 antibody (1:150 dilution *v*/*v*; Santa Cruz Biotechnology, Santa Cruz, CA, USA). The slices were then washed with PBS 1X and incubated in the dark with the fluorescein isothiocyanate-conjugated anti-rabbit (Abcam, Cambridge, UK). The nuclei were stained by Hoechst. Sections were analyzed with a microscope (Nikon Eclipse 80i), and images were captured by a high-resolution digital camera (Nikon Digital Sight DS-U1).

### 4.9. Protein Extraction and ZO-1 and Occludin Immunoblot Analysis

Colonic tissue isolated on the 3rd and 7th day after colitis induction or bioptic samples were processed for Western blot analysis. Briefly, the samples were homogenized in an ice-cold hypotonic lysis buffer with (10 mM of 4-(2-hydroxyethyl)-1-piperazineethanesulfonic acid (HEPES), 1.5 mM of MgCl_2_, 10 mM of KCl, 0.5 mM of phenyl-methyl-sulphonylfluoride, 1.5 mg/mL of the soybean trypsin inhibitor, 7 mg/mL of pepstatin A, 5 mg/mL of leupeptin, 0.1 mM of benzamidine and 0.5 mM of dithiothreitol (DTT)). The resulting cytosolic extracts were mixed with a non-reducing gel loading buffer (50 mM Tris (hydroxymethyl) aminomethane (Tris), 10% sodium-dodecylsulfate (SDS), 10% glycerol, 2 mg bromophenol/mL) at a 1:1 ratio, and then boiled for 3 min followed by centrifugation at 10,000× *g* for 10 min. The protein concentration was determined using the Bradford assay, and equivalent amounts (50 μg) of each homogenate underwent electrophoresis through a polyacrylamide mini gel. Proteins were transferred to nitrocellulose membranes that were saturated via incubation with 10% non-fat dry milk in PBS 1X overnight at 4 °C before incubating with rabbit polyclonal anti-ZO-1, rabbit monoclonal anti-occludin (Abcam, Cambridge, UK) or mouse monoclonal anti-β-actin (Santa Cruz Biotechnology, CA, USA) for 2 h at room temperature (RT). Membranes were then incubated with the specific secondary antibodies conjugated to horseradish peroxidase (HRP) (Dako, Milan, Italy). Immune complexes were revealed by enhanced chemiluminescence detection reagents, and immunoreactive protein bands were then visualized, scanned, and densitometrically analyzed with ChemiDoc XRS+ apparatus (Bio-Rad Laboratories S.r.l. Segrate, Milano, Italy). The results were expressed as the % of protein expression and normalized on the expression of the housekeeping protein β-actin for mouse proteins.

### 4.10. Plasma Collection and Adelmidrol Quantification in the Blood

Mouse blood samples were obtained via a cardiac puncture during anesthesia for the endoscopic procedure. Blood samples were, thus, collected in 5% EDTA vials immediately prior to sacrifice to determine the adelmidrol level in the blood following its intrarectal administration. The adelmidrol level was measured according to the procedure described by D’Amico et al. (2020) [33], using LC-MS/MS (Agilent Technologies G6470A) as a function of time. Briefly, an amount of 100 μL of the plasma samples was diluted in 900 μL of acetonitrile, and the relative absorption rate of Adelmidrol in the plasma was quantified using a stock solution of adelmidrol in methanol. The five-point calibration curve was prepared by dilution in acetonitrile from the stock solution as previously described [31].

### 4.11. ELISA Quantification of LPS (Endotoxemia), IL-1β, IL-6, and TNF-α in the Mouse Plasma and Human Bioptic Samples

Enzyme-linked immunosorbent assay (ELISA) kits were used to quantify both LPS (Chondrex Inc., Woodinville, WA, USA), IL-1β, IL-6, and TNF-α (Thermo Fisher Scientific, MA, USA) in the plasma of collected mice blood samples and homogenized human bioptic samples according to the relative manufacturer’s instructions.

### 4.12. Myeloperoxidase Activity

Myeloperoxidase (MPO) activity was evaluated in colonic tissues to determine the extent of neutrophil infiltration and activation, as previously described [34]. After removal, mouse colonic tissues or human-cultured bioptic samples were rinsed in a cold saline solution, then homogenized in a solution containing 0.5% hexadecyltrimethylammonium bromide (Sigma-Aldrich) dissolved in 10 mM of a potassium phosphate buffer and centrifuged for 30 min at 20,000× *g* at 37 °C. An aliquot of the supernatant was mixed with a solution of tetramethylbenzidine (1.6 mM; Sigma-Aldrich) and 0.1 mM of hydrogen peroxide (Sigma-Aldrich). The absorbance was then spectrophotometrically measured at 650 nm. MPO activity was determined as the amount of the enzyme degrading 1 mmol/min of peroxide at 37 °C and was expressed in milliunits per 100 mL of the homogenized sample.

### 4.13. Lipid Peroxidation Assay

Malonyl dialdehyde (MDA) was measured via the thiobarbituric acid colorimetric assay according to the method described by Esposito et al. (2012) [35] in isolated colonic tissues on the 3rd and 7th day after DNBS challenge and in ex vivo human-cultured biopsies. Briefly, 1 mL of 10% (*w*/*v*) trichloroacetic acid was added to 450 μL of the tissue lysate. After centrifugation, 1.3 mL 0.5% (*w*/*v*) of thiobarbituric acid was added, and the mixture was heated at 80 °C for 20 min. After cooling, MDA formation was recorded (absorbance 530 nm and absorbance 550 nm) in a Perkin Elmer (Waltham, MA, USA) spectrofluorimeter, and the results were presented as ng MDA/mL.

### 4.14. PEA Level Quantification

The extraction and analysis of PEA levels were performed according to [36], with slight modifications. Mouse colonic tissues or human-cultured bioptic samples were firstly lysed and then evaporated under a nitrogen stream. Residues were suspended in an extraction solution, ultracentrifuged (14,000 rpm, 4 °C, 5 min), and the supernatant was injected for mass spectrometry analysis. Analyses were run on a Jasco Extrema LC-4000 system (Jasco Inc., Easton, MD, USA) coupled to an Advion Expression mass spectrometer (Advion Inc., Ithaca, NY, USA) and equipped with an electrospray (ESI) source. Mass spectra were recorded in the positive SIM mode. The capillary voltage was set at +180 V, the spray voltage was set at 3 kV, the source voltage offset was set at +20 V, and the capillary temperature was set at 250 °C. The chromatographic separation was performed on the analytical column Kinetex C18 (150 × 4.6 mm, id. 3 μm, 100 Å) and a security guard column, both supplied by Phenomenex (Torrance, CA, USA). The analyses were performed at a flow rate of 0.3 mL/min, with solvent A (water containing 2 mM ammonium acetate) and solvent B (methanol containing 2 mM ammonium acetate and 0.1% formic acid). Elution was performed according to the following linear gradient: 15% B for 0.5 min, 15–70% B from 0.5 to 2.5 min, 7–99% B from 2.5 to 4.0 min, and held at 99% B from 4.0 to 8.0 min. From 8 to 11.50 min, the column was equilibrated to 15% B and conditioned from 11.5 to 15.0 at 15% B. The injection volume was 10 μL, and the column temperature was fixed at 40 °C. For quantitative analysis, standard curves of PEA (Sigma-Aldrich, Milan, Italy) were prepared over a concentration range of 0.0001–10 ppm with six different concentration levels, and duplicate injections were prepared at each level. All data were collected and processed using JASCO Chrom NAV (version 2.02.04) and Advion Data Express (4.0.13.8).

### 4.15. Statistical Analysis

The results were expressed as the mean percentage. Statistical analysis was performed using parametric one-way analysis of variance (ANOVA), and multiple comparisons were performed using Bonferroni’s post hoc test. *p*-values < 0.05 were considered statistically significant. Data were analyzed using Graphpad Prism 9 and ImageJ 1.53 software.

## Figures and Tables

**Table 1:** Quantification of adelmidrol.

Time (Hours after Intrarectal Administration)	Level of Quantification (LOQ) [ng/mL] in the Plasma
0	<71.4
72	<71.4
168	<71.4

## Data Availability

The data presented in this study are available on request from the corresponding author.
